# Prevalence of Premenstrual Syndrome and Its Association With Dietary Inflammatory Index Among Young Females: A Cross-Sectional Study

**DOI:** 10.1155/jnme/4189297

**Published:** 2025-09-11

**Authors:** MoezAlIslam Faris, Mona Hashim, Dana N. Abdelrahim, Falak Zeb, Iftikhar Alam, Alya Salim Alzaabi, Fatima Khalil Alhamadi, Noor Akram Issa, Hamda Sharif Al Ali, Maya Mohammad AlSaffarini

**Affiliations:** ^1^Department of Clinical Nutrition and Dietetics, Faculty of Allied Medical Sciences, Applied Science Private University, Amman, Jordan; ^2^Department of Clinical Nutrition and Dietetics, College of Health Sciences, University of Sharjah, Sharjah, UAE; ^3^Research Institute of Medical and Health Sciences (RIMHS), University of Sharjah, Sharjah, UAE; ^4^Department of Human Nutrition and Dietetics, Bacha Khan University, Charsadda, Pakistan

**Keywords:** adiposity, dietary habits, lifestyle factors, premenstrual syndrome (PMS), reproductive health

## Abstract

This study investigated the prevalence and severity of premenstrual syndrome (PMS) among adult females and its relationship with the dietary inflammatory index (DII), which measures the inflammatory potential of a person's diet. The study employed a cross-sectional design, with participants recruited through convenience sampling. A structured questionnaire, including the Arabic Premenstrual Syndrome Scale, was used to assess the prevalence and severity of PMS. A self-administered food frequency questionnaire was used to assess dietary intakes and depict the DII of the participant's intakes. A total of 305 adult females participated; of these, 93% reported at least one PMS symptom, with a prevalence of 33.7% for PMS, primarily characterized by mild to moderate symptoms. The mean DII score was 2.52 ± 6.28, indicating a generally proinflammatory diet among participants. Multiple logistic regression analyses revealed that higher DII scores, particularly in Tertile 3, were significantly associated with increased PMS severity (*p*=0.001). These findings highlight the importance of dietary modifications that aim to reduce inflammation as a potential strategy for mitigating the severity of PMS. Future research should focus on longitudinal studies to establish causality and explore the effectiveness of anti-inflammatory dietary interventions in managing PMS symptoms.

## 1. Introduction

Premenstrual syndrome (PMS) is a recurring disorder in the late luteal phase of the menstrual cycle, marked by emotional, physical, and psychological symptoms [1]. These symptoms, occurring from a week before to a few days after menstruation, significantly affect women's quality of life (QoL) [1, 2]. Further, the severity of PMS varies based on physiological, psychosocial, and hormonal variables [1]. It has been reported that PMS impedes the development of female adolescents and young adults attempting to reach developmental milestones. PMS may result in decreased health and work-related QoL [3, 4], decreased occupational productivity [5], interference with interpersonal relationships and daily life [6, 7], and increased reliance on specialized healthcare [8, 9]. In addition, PMS may be significantly associated with academic performance impairment [10, 11], diminish the QoL at work [12, 13], increase the risk of hypertension [14, 15], and impair the daily activities of collegiate athletes and athletic performance [16]. Moreover, PMS has been linked to alterations in emotional and cognitive processes [17].

As with many other syndromes, PMS is caused by the interaction of multiple genetic (racial and ethnicity-related) [18] and lifestyle behaviors [18–21], with dietary factors being the most influential [21]. There is evidence in the literature that links ethnicity and/or cultural context to PMS and other premenstrual illnesses.

Young females are among those who are most susceptible to PMS. This group has a high prevalence of PMS, which negatively influences their QoL and academic performance [13, 18–25]. Each country reports a different prevalence of PMS in adult females; for instance, 65% in Egypt [26], 72.1%–91.8% in Turkey [18, 27], 39.4%–56.9% in Iran [23, 28], 33.82% in China [10], 39.9% in Taiwan [19], 37% in Ethiopia [20], 79% in Japan [21], 80% in Pakistan [22], and 89.5% in South Korea [25]. The observed geographical variation in the prevalence of PMS could be explained by differences in the dietary, lifestyle, and genetic makeup conditions of the young adult females under study. Furthermore, community-based customs surrounding menstruation have a significant impact in this regard [29]. Differences in study methodology, such as the use of controls for confounding variables [28], assessment methods, and independent variables, may also explain variations in PMS prevalence. Furthermore, differences in how specific physical and psychological experiences are constructed or understood in society as a disease connected to the reproductive system may also account for cultural differences in PMS prevalence [30, 31].

Female adults in the Gulf Cooperation Council (GCC), the United Arab Emirates (UAE), and other Islamic countries have adopted community- and culture-based health beliefs, attitudes, and practices related to menstruation and puberty [18–21]. They have also adopted various dietary and lifestyle practices, as well as remedial methods to ease premenstrual and menstrual symptoms [21, 22]. These include the use of analgesics and vitamins as a part of a healthy diet [21], as well as an increase in the consumption of vegetables and fruits, personal hygiene, heated bathing, and exercise [22].

A scoring system called the dietary inflammatory index (DII) was developed to measure the total impact of food on the body's inflammatory state [19, 20]. The observed strong negative connection between the DII and total energy intake in multiple groups led to the development of the energy-adjusted DII (E-DII). E-DII is a measure that assesses the inflammatory potential of an individual's diet, adjusted for total energy intake to allow for more accurate comparisons across different dietary patterns [32]. Both the DII and the E-DII aim to assess a diet's quality according to its capacity to cause inflammation in the body by analyzing the intakes of different vital nutrients and frequently consumed bioactive phytochemicals [19].

The DII and E-DII are scored and scaled similarly, with the exception that the total energy consumed is included in the denominator of the E-DII; as a result, the scores are equivalent across studies. The development of the DII and E-DII was grounded in published research on a set of dietary-related inflammatory indicators, including TNF-α, CRP, IL-1, IL-6, IL-4, and IL-10 [33]. A low DII score indicates an anti-inflammatory diet, whereas a high DII/E-DII score suggests a high consumption of proinflammatory compounds. Validation of the DII/E-DII is done against a set of inflammatory indicators, such as homocysteine, TNF-, IL-6, and CRP [34]. It has been reported that high DII/E-DII scores are associated with anxiety, cognitive decline, and severe mental disorders and thus proposed to be associated with PMS, as it comprises mental and pain (progesterone-inducing effects) aspects. In a previous study examining female adolescents in the UAE, it was discovered that PMS was prevalent and had a detrimental impact on their educational, social, and emotional well-being, highlighting a significant public health concern [11]. Nevertheless, there has been a shortage of research investigating the prevalence of PMS and its association with the DII among adult females. Therefore, this study aims to determine the prevalence of PMS among young females in Sharjah/UAE and to investigate its association with the DII. By examining the dietary inflammatory potential in this population, the research aims to identify modifiable risk factors that could inform preventive strategies and nutritional interventions to mitigate the severity and prevalence of PMS.

## 2. Participants and Methods

### 2.1. Design and Setting

This quantitative, cross-sectional study was conducted at the University of Sharjah (UOS), Sharjah City/UAE, from February to May 2023.

### 2.2. Research Consent and Permissions

This investigation was approved by the UOS Research Ethics Committee (REC/16/02/07/S), and the female Deanship office at the UOS granted official permission. Before providing their written informed consent, participants received thorough verbal explanations of the study's goals and purposes. The participants received information guaranteeing that the data they gathered would be strictly confidential and would not be misused. Because of privacy concerns, the names and addresses of participants were not collected. No financial or nonfinancial incentives were provided to encourage participation. Premenopausal adult females in the UOS and surrounding areas, including faculty and employee residences, who had regular menstrual cycles for at least the previous 3 months, were eager to participate in the study and were able to provide informed consent that met the eligibility requirements. Pregnancy: a history of recognized chronic illnesses, such as diabetes, sickle cell anemia, and other ailments, mental health problems; irregular menstrual cycles; and being younger than 18 or older than 45 were among the exclusion criteria. Even if one of the factors influencing the occurrence and severity of PMS is oral contraceptives, which represent exogenous hormones that may influence PMS [20, 21], no questions regarding oral contraceptives were asked because sociocultural and religious norms discourage their use among adults, and nonmarried females [21]. Due to cultural constraints, the investigators were unable to discuss the use of oral contraceptives, even with married women. Asking study participants about their use of contraceptives is deemed sensitive and unpleasant because the majority (87%) were unmarried. In Islamic and Arabic nations, including the UAE, it is illegal and culturally taboo to perform sexual activity before marriage.

### 2.3. Sample Size and Sampling Procedure

Approximately 8700 adult female students, faculty members, and employees from UOS campuses, female residences, and affiliated institutions were invited to participate in the cross-sectional survey. The invitation letter stated the collection site, and at least one reminder was provided to the adult females to participate. Furthermore, a campaign to recruit participants was promoted through the university website, social media platforms (Facebook, Instagram, and Twitter), and direct outreach to adult females in the UOS. Those who expressed their willingness to participate and visited the designated data-collecting site inside the UOS were informed about the study's goals and protocol before being given the consent form to sign. Once the consent was signed, the eligible participant was given a tablet with access to the online questionnaire.

The recruited participants represented different nationalities, including nonlocal GCC residents, Arab non-GCC expatriates, non-Arab non-GCC residents, and local Emirati women.

With a 30% nonresponse assumption and a single proportion for a finite population, the sample size was determined. Stata 13.1 was used to determine the minimal number of participants required to establish a prevalence proportion of at least 30% using a two-sided alpha of 0.05% and 80% power. Furthermore, the sample size estimates were based on the ability to detect associations between PMS and anthropometric and lifestyle characteristics. To estimate the sample size, a small effect size of approximately 0.5 Cohen's d, a power or type II error of 0.8 (80%), and a level of significance or type I error of 0.05 (5%) were chosen. Based on the resulting sample size estimates, it was determined that a sample size of 180–220 would be adequate for the analyses.

### 2.4. Data Collection Tools

A self-administered, semi-structured questionnaire was utilized to gather data. A list of PMS symptoms from multiple sources, along with pertinent demographic data regarding the study participants' health profiles, was included in the questionnaire. Before the study began, a pilot study was conducted to gather data on 25 UOS females (8.3% of the intended sample size of 305 adult females). This study aimed to standardize techniques, assess the validity and repeatability of the data collection instrument, and evaluate the performance of the data collectors. It also aimed to identify any problems with the data collection tools. Before being finalized, the data were collected on the day of collection and checked for accuracy and completeness. Before data entry, the principal investigator precisely coded and altered the data. To ensure data quality, 5% of the data were re-entered prior to data analysis. The researchers also gathered information on dietary habits, lifestyle factors (such as smoking and physical activity), anthropometric data (including weight, height, body mass index [BMI], and waist circumference [WC]), and demographics (including age, marital status, education, and nationality). Assessment of dietary intake was performed using a validated food frequency questionnaire (FFQ), with Cronbach's alpha and McDonald's omega coefficients exceeding 0.9 [19]. A complete description of the FFQ is published elsewhere [21, 22].

Food models and related images were utilized to standardize data collection further and develop a common unit of measurement for portions. In brief, the FFQ assessed the intake of 54 items from five major food groups over the preceding four weeks. According to a recent systematic review and meta-analysis, using FFQs (usually within the last month) is a reliable method in nutritional epidemiology research [23]. Using ESHA's Food Processor® nutrition analysis software, dietary analysis of the FFQ was carried out, and DII/E-DII was then estimated. We utilized the up to thirty-item meal parameters found in the DII and E-DII. Dietary components required for DII calculations were available in this investigation. Total caloric intake (kcal), caffeine-containing beverages and tea (g), protein (g), carbohydrate (g), fiber (g), sugar (g), total fat (g), cholesterol (mg), saturated fat (g), MUFA (g), PUFA (g), sodium (Na), calcium (Ca), iron (mg), vitamin C (mg), vitamin D (μg), vitamin E (mg), thiamin (vitamin B_1_, mg), riboflavin (vitamin B_2_, mg), vitamin B_6_ (mg), folate (μg), vitamin B_12_ (μg), vitamin A (RE), rosemary (mg), thyme/oregano (mg), garlic (g), onion (g), black pepper (g), turmeric (mg), saffron (g), and ginger (g) are some of these [20, 35].

The worldwide standard database was compared with each of the individual food intake metrics that make up the DII. A detailed description of the DII and its calculating algorithm can be found elsewhere [19, 20]. Data on the individuals' food intake were gathered using the FFQ to estimate DII. After that, the following actions were done: Initially, a robust estimate of the mean and standard deviation (SD) for each food parameter was obtained by correlating the ingestion of food data with a globally representative database that was indicative of a particular location. Second, *Z*-score normalization was employed to estimate each participant's exposure to the global mean using the standard *Z*-score procedure. Thirdly, to lessen the impact of right skewing, the *Z* scores were converted into proportions. Fourth, to create a normal distribution with values centered on 0 (*neutral*) and limited by 1 (*highest anti-inflammatory*) and +1 (*most proinflammatory*), these proportions were multiplied by 2 and then subtracted by 1. Fifth, to determine the parameter-specific DII score for each food item, the centered proportion value was multiplied by the inflammatory impact score that corresponded to it. Sixth, the sum of the DII ratings for every meal parameter was used to calculate the overall DII score [20].

The E-DII for each participant in this study was obtained using a unique referent database of energy-adjusted parameter-specific scores based on data from the same countries used to calculate the DII. More details on E-DII are available in other sources [19]. Participants were sorted into tertiles based on E-DII scores. Tertiles of E-DII scores were computed based on the distribution of E-DII among controls according to the following ranges: Tertile 1 ≤ −4.719, Tertile 2 between −4.720 and 15.756, and Tertile 3 ≥ 15.760.

Every participant had their anthropometric measurements taken, including height, which was measured using a stadiometer (Seca 220, Hamburg, Germany) and reported to the nearest 0.01 m. Body composition was examined using the bioelectrical impedance (BIA) method (InBody 230 model, MW160, Seoul, Korea). BMI (kg/m^2^) was calculated and classified according to World Health Organization guidelines, with participants categorized as having a normal weight if their BMI was less than 25.0 kg/m^2^ and as being overweight or obese if their BMI was 25.0 kg/m^2^ or greater. Body weight was measured to the nearest 0.1 kg. According to the Centers for Disease Control and Prevention, anthropometric measures were taken [35]. Measurements for BIA were taken in compliance with the manufacturer's instructions. Before consuming breakfast and after taking off any metallic accessories, each student was measured. Before beginning the BIA testing, every participant was asked about pacemakers and metal implants.

A four-point Likert scale is used in the PMS symptoms questionnaire to indicate the severity of symptoms, ranging from *none* to *severe*. In the Arabic Premenstrual Syndrome Scale (APMSS) survey [36]. Respondents are asked if, throughout the preceding 3 months, they experienced particular PMS symptoms. The questionnaire consists of 25 questions, divided into three categories: behavioral, physical, and psychological symptoms.

Psychological symptoms include depression, hopelessness, guilt feelings, anxiety, worry, emotional lability, increased sensitivity to others, hostility, difficulties concentrating, quickly getting agitated or annoyed, lack of interest, difficulty maintaining control, and a sense of overload. Physical symptoms include headache; muscle, joint, or back discomfort; acne; lethargy; exhaustion; decreased energy; increased hunger; a craving for certain foods; hypersomnia; insomnia; breast soreness; breast engorgement; and weight gain. Behavioral symptoms are those that cause problems in relationships, at work, or in the classroom. It took around 15 minutes to score and analyze the APMSS findings. The severity of the symptoms was expressed in four terms: none, mild, moderate, and severe. It focuses only on the symptoms that participants experienced 1 week before menstruation and a few days into menstruation in the last 3 months. The online survey was distributed by email and social media platforms (e.g., WhatsApp). The validity and reliability of the APMSS were previously studied by Algahtani and Jahrami [37] to be suitable for Arabic-speaking participants, with a relatively high Cronbach's alpha value of 0.910.

### 2.5. Data Analysis

The Strengthening the Reporting of Observational Studies in Epidemiology (STROBE) criteria [13] were followed in reporting the analyses. SPSS Version 29 was used to code, input, and analyze the questionnaires. Point estimates for the sample were computed using descriptive statistics, such as the mean and SD for continuous variables (e.g., weight, height, BMI, and nutrient intake) and frequencies and percentages for categorical variables (e.g., PMS levels, severity of symptoms, and sociodemographic characteristics). Before starting the studies, the data were formally examined for normality using the Shapiro–Wilk test and visually verified. Using PMS prevalence and severity as the outcome variable and sociodemographics as independent variables, multinomial logistic regression analysis was employed to evaluate the impact of the E-DII. Tertiles were fitted to the E-DII scores. The models underwent adjustments based on age, BMI, marital status, educational attainment, and nationality. To account for the same factors, linear multiple regression analysis also employed E-DII as a continuous score. For all subsequent analyses, E-DII was utilized, as it accounts for each participant's overall energy consumption.

## 3. Results

A total of 305 premenopausal adult females completed the APMSS (Table 1). The age range of the participants was 20–45 years, with a mean age of 23.56 ± 7.81 years. Approximately 96% of the participants were females under the age of 40 years. The mean BMI was 24.09 ± 5.24 kg/m^2^, the mean height was 160.08 ± 6.46 cm, and the mean weight was 61.83 ± 14.29 kg. The vast majority of participants (86.6%) were unmarried. More than half of the participants (approximately 56%) hold a university degree. Approximately 39% have a WC exceeding 76 cm (Table 1).

Table 2 represents the lifestyle characteristics of the participants. It shows that 92.8% of them never smoked, while only 15.1% of the participants did not engage in physical activity. Additionally, 51.8% practice physical activity for less than 30 min per day, and 33.1% practice physical activity for more than 30 min per day. More than half (64.9%) of the participants had daily sun exposure of fewer than 15 min. Additionally, 72.5% of participants wore an abaya, a traditional loose dress that covers the entire body, and 59.0% of them lived in villas.

Table 3 summarizes the dietary characteristics of 305 participants, detailing their daily intake of nutrients and foods. On average, participants consumed 3540.81 ± 1919.67 kcal of energy. Protein intake averaged 137.06 ± 77.69 g, while carbohydrate was 398.35 ± 212.48 g, and total fat was 160.88 ± 93.34 g per day. Cholesterol intake was 563.11 ± 385.38 mg, and participants consumed 68.58 ± 38.92 g of saturated fat, 55.73 ± 35.17 g of monounsaturated fat (MUFA), and 25.66 ± 15.10 g of polyunsaturated fat (PUFA). Sodium intake averaged 3804.15 ± 2211.87 mg, with vitamin C at 295.50 ± 176.60 mg and calcium at 1778.32 ± 1038.41 mg. The average intake of iron was 27.23 ± 16.11 mg, vitamin D was 12.69 ± 9.40 μg, and vitamin E was 15.01 ± 9.18 mg. Other vitamins and minerals included thiamin (B_1_) (4.79 ± 2.71 mg), riboflavin (B_2_) (10.88 ± 6.39 mg), pyridoxine (B_6_) (3.48 ± 2.08 mg), folate (B_9_) (529.15 ± 349.56 μg), and cobalamin (B_12_) (10.49 ± 14.73 μg). Dietary fiber was consumed at an average of 32.93 ± 22.26 g per day, with total sugars at 140.89 ± 77.21 g. Participants' intake of vitamin A was 2029.45 ± 1822.46 μg. The table also details the consumption of specific herbs and spices, including rosemary (2.46 ± 2.78 g), thyme/oregano (1.98 ± 2.74 g), garlic (4.35 ± 3.40 g), onion (11.53 ± 8.93 g), black pepper (3.97 ± 3.01 g), turmeric (3.01 ± 2.85 g), saffron (2.14 ± 3.23 g), and ginger (2.48 ± 2.60 g).

Table 4 presents the prevalence and severity of PMS symptoms among the 305 participants. Symptoms were categorized into four levels of severity: never, mild, moderate, and severe. Depressed mood was reported as mild by 44.6% of participants, moderate by 29.5%, and severe by 7.2%. The feelings of hopelessness were mild in 37.7%, moderate in 22.3%, and severe in 5.6% of participants. Anxiety or worry was mild in 35.4%, moderate in 37.4%, and severe in 14.8%. Affective ability, or mood swings, was mild in 35.1% and moderate in 27.9%, with 9.8% experiencing it severely.

Participants also reported increased sensitivity toward others (mild in 38.0%, moderate in 34.8%, severe in 13.1%) and feelings of anger (mild in 39.0%, moderate in 29.8%, severe in 17.7%). Easily irritated or agitated feelings were mild in 37.4%, moderate in 35.7%, and severe in 13.8%. A lack of interest was reported as mild by 39.3% and moderate by 30.2%, with 8.5% experiencing it severely. Other common symptoms included difficulty concentrating (mild in 40.7%, moderate in 28.9%, severe in 10.8%), feeling overwhelmed (mild in 32.5%, moderate in 38.4%, severe in 19.0%), lethargy/fatigue (mild in 33.4%, moderate in 39.3%, severe in 19.0%), and increased appetite (mild in 33.8%, moderate in 30.2%, severe in 12.5%). Cravings for certain foods were mild in 36.1% and moderate in 29.8%, with 17.7% experiencing them severely.

Physical symptoms such as breast tenderness, headaches, and muscle or joint pain were also prevalent, with varying degrees of severity. Cumulative psychological symptoms were mild in 55.1%, moderate in 36.4%, and severe in 1.0%. Cumulative physiological symptoms were mild in 63.3% and moderate in 31.8%, with only 1.3% experiencing them severely. Behavioral symptoms were reported as mild by 42.6%, moderate by 24.6%, and severe by 2.6%. Overall, 65.2% of participants experienced mild total PMS symptoms, 25.2% moderate, and only 0.3% severe.

Table 5 presents the odds ratios (ORs) and confidence intervals (CIs) for the tertiles of E-DII in relation to the dietary habits of patients with PMS. The participants were divided into three tertiles based on their E-DII scores: Tertile 1 (E-DII ≤ −4.719), Tertile 2 (E-DII between −4.720 and 15.756), and Tertile 3 (E-DII ≥ 15.760). The participants were divided into three tertiles, with 102 participants in Tertile 1, 102 in Tertile 2, and 101 in Tertile 3, all of whom reported experiencing PMS. The reference group was Tertile 1, with an OR of 1.0. Compared to Tertile 1, the odds of PMS in Tertile 2 were 1.77, with a 95% CI of 0.425–1.418. For Tertile 3, the OR was higher at 2.151, but the CI of 0.831–5.564 indicates a wide range of uncertainty. When analyzed as a continuous variable, the E-DII had an OR of 1.639, with a CI range of −0.107 to 0.121, indicating no significant association.

## 4. Discussion

This study aims to determine the prevalence and severity of PMS among adult females in Sharjah, UAE, and to examine its associations with the DII. Results indicated that 93% of participants reported at least one PMS symptom during their menstrual period. The prevalence of PMS was 33.7%, with mild-to-moderate symptoms being the most frequently reported. In addition, analysis of the DII revealed a mean ± SD of 2.52 ± 6.28, indicating an overall proinflammatory diet that may trigger PMS symptoms. A positive DII value suggests a diet that can promote bodily inflammation.

The health of female university students, particularly in relation to PMS, has been a topic of interest to researchers in the UAE. The prevalence rate of PMS ranged from 35.3% [8]. The prevalence rates of psychological, physical, and behavioral symptoms were 83.0%, 79.4%, and 76.6%, respectively [38]. While a recent study [37] found that 78.9% of participants experienced premenstrual tension syndrome, a subsequent study [38] reported that 90% of participants experienced a negative impact on their academic performance, with paying attention in class being the aspect most significantly affected. Diets rich in cruciferous vegetables and fruits were significant factors associated with a decrease in PMS [8, 38]. In both studies, high-calorie, high-fat, high-sugar, and high-sodium food consumption was identified as a strong risk factor for PMS.

Based on the results of this research, we can confirm that our initial hypothesis was correct: DII/E-DII is directly associated with the severity of PMS, and consuming a diet that increases the DII/E-DII value is likely to exacerbate the severity of PMS symptoms in adult females. Further, the luteal phase of the menstrual cycle is characterized by a proinflammatory state, with elevated levels of proinflammatory cytokines observed compared to the follicular phase. This includes an increase in type-1 proinflammatory signals and cytokines such as IL-1β and TNF-α during the luteal phase [39].

Studies have demonstrated that certain dietary components in foods are associated with increasing the DII/E-DII (such as hydrogenated oil, high-fat foods, and salty, high-sugar foods) by increasing the C-reactive protein (CRP) [40]. In addition, food types are associated with decreasing DII/E-DII (such as olive oil, nuts, fruits, and vegetables) by providing the anti-inflammatory potential to the body [41] by reducing the concentration of interleukin (IL)-6 and CRP [42, 43]. Anti-inflammatory foods possess this potential, in part, by providing significant concentrations of polyphenols. These polyphenols inhibit phospholipase A2, cyclooxygenase, and lipoxygenase, thereby reducing the production of prostaglandins and leukotrienes and mitigating chronic inflammation [44].

In more detail, the biological mechanisms underlying the influence of anti-inflammatory factors on symptoms of PMS are associated with systemic inflammation, as reflected by increased proinflammatory cytokines (IL-6, TNF-α, or CRP) in the luteal phase [45]. Proinflammatory food components, such as refined sugar, *trans* fatty acids, and processed foods, can exacerbate inflammation, potentially leading to an increase in PMS symptoms. On the other hand, omega-3 fatty acids found in fatty fish, walnuts, and flaxseeds help inhibit inflammation by suppressing the synthesis of prostaglandins, a key contributor to PMS-related cramps and pain. Omega-3 fatty acids also modulate immune functions by reducing IL-6 and TNF-α levels, thus lessening mood disturbances. Likewise, antioxidants in fruits, vegetables, green tea, and dark chocolate counteract oxidative stress and inflammation. Specifically, polyphenols, such as flavonoids in berries and curcumin in turmeric, suppress the NF-κB pathway, thereby lowering inflammatory cytokine expression. Additionally, magnesium, found in nuts, leafy greens, and seeds, acts as a natural muscle relaxant, helping to prevent PMS-associated cramps and headaches. Vitamin D, a key regulator of immune modulation, has been shown to reduce levels of CRP, potentially improving mood and pain perception. Another critical nutritional component is fiber, found in whole grains, legumes, and vegetables, which helps maintain gut microbiota balance, reduces systemic inflammation, and helps balance hormone fluctuations associated with PMS. Short-chain fatty acids (SCFAs) produced by gut microbes have anti-inflammatory effects that may alleviate PMS symptoms [46]. Subsequent studies must clearly associate DII scores with these biological processes, illustrating how a lower DII score (reflecting an anti-inflammatory diet) is related to a decrease in PMS severity. Studies must also examine the relationships between DII scores and inflammatory biomarkers (CRP, IL-6, and TNF-α) to confirm these postulated pathways.

Experimental research has demonstrated that concentrations of circulating inflammatory markers, including proinflammatory cytokines, increase around ovulation and reach their peak during menstruation. Therefore, it is postulated that cytokines adversely impact stress response routes and neurotransmitter function in psychological derangements that share similar characteristics with PMS [47, 48].

When observing the macronutrient intakes of the participants, a high intake of dietary fats (41% of total energy intake) was detected (data not shown). This high intake of fats may have been a major factor contributing to the increase in the severity of PMS in a large portion of the studied population. Lower fat intake, anticipated by “no fast-food consumption” among university students in Dubai, was among the significant detrimental factors in decreasing PMS [38]. Existing literature suggests that dietary fats may influence hormone and cytokine levels in women, potentially triggering inflammation. Changes in hormones can play a significant role in the etiopathogenesis of PMS and contribute to its increased severity [48, 49].

A recent randomized controlled trial was conducted to find out how well pomegranate use affects QoL and PMS symptoms [50]. It was observed that symptoms decreased significantly in the intervention group after the intervention (*p* < 0.05). Pomegranate is a rich food source with anti-inflammatory, antioxidant, antidepressant, and phytoestrogenic properties. Estrogens are a factor that regulates emotions, especially in women. Fluctuations in estrogen levels are known to produce anxiety and depressive symptoms [51]. Especially in reducing PMS symptoms, and may also improve social QoL [50].

This study focused on PMS symptoms during the menstrual period, and several additional reasons support the results. First, nutrient imbalances, including nutritional deficiencies in essential vitamins, minerals, and other nutrients, can contribute to PMS symptoms. For example, inadequate intake of certain nutrients, such as calcium, magnesium, vitamin D, and vitamin B_6_, has been linked to an increase in PMS symptoms. Additionally, some women turn to eating sugar-rich foods during PMS, which can lead to rapid blood sugar fluctuations.

In a study among Saudi women attending health centers in Riyadh, which investigated diet, psychological distress, and lifestyle factors in relation to premenstrual symptoms, a significant association was found between higher sugar intake and the presence of anxiety/mood symptoms as well as other physical symptoms, with women in the higher sugar intake group being 1.53 times more likely to experience these symptoms (95% CI: 1.07–2.19). In this regard, a recent systematic review investigated the effects of nutritional interventions on PMS and showed some windows for decreasing psychological symptoms. The review concluded that high consumption of bread and snacks, as well as low adherence to a Mediterranean diet, was associated with a higher risk of PMS. Well-known evidence shows that the Mediterranean diet is characterized by high consumption of whole grains, nuts, olive oil, legumes, fruits, and vegetables, all of which are rich in anti-inflammatory factors, vitamins, antioxidants, polyphenols, and unsaturated fats that may reduce symptoms of PMS by attenuating oxidative stress and inflammation that predispose the development of PMS [48, 52]. A postulated protective property of the MD on PMS is attributed to its high content of omega-3 fatty acids, which is linked to its ability to release beta-endorphin into the hypothalamus, leading to a reduction in pain and depressive-like symptoms of PMS [53]. In vitro and in vivo experimental studies have demonstrated that bioactive molecules, such as curcumin, allicin, anethole, thymoquinone, gamma-linoleic acid, and various other compounds, possess not only antioxidant and anti-inflammatory properties but also multiple additional activities, including serotonergic, antidepressant, sedative, and analgesic effects [48]. Embracing a balanced diet that includes complex carbohydrate, protein, and healthy fats can help stabilize blood glucose levels and minimize PMS symptoms [54]. When comparing the aforementioned results with the existing literature, no findings were found that contradicted the results obtained in this research [54].

### 4.1. Strengths, Limitations, and Suggestions

Our study is the first to examine the association between PMS and the DII among young females in the UAE and GCC, providing novel, region-specific data. Using a validated FFQ and the Arabic PMS Scale enhances the cultural relevance and objectivity of our assessments. The significant positive association found between higher DII scores and increased PMS severity highlights a modifiable dietary factor that could inform intervention strategies.

However, the study's cross-sectional design limits causal inferences, and reliance on self-reported dietary and symptom data introduces recall bias. The convenience sampling limits generalizability beyond college students of similar age and socioeconomic background. Additionally, potential residual confounding, wide CIs, and the absence of standardized measures of PMS severity suggest that further research, including larger, multi-center, prospective studies, is needed to confirm and expand upon these findings.

Future studies should consider employing longitudinal designs to establish the causal relationship between dietary inflammatory potential and the severity of PMS. Including larger, more diverse populations across different age groups and regions within the GCC and the UAE will improve the generalizability of findings. Utilizing standardized and comprehensive tools for assessing PMS symptoms and severity can enhance accuracy and reliability. Additionally, incorporating objective dietary assessment methods, such as biomarkers, alongside self-reports may reduce recall bias. Multi-center collaborations and randomized controlled trials focusing on dietary interventions, such as anti-inflammatory diets, are also recommended to better understand and confirm the potential for dietary modification to alleviate PMS symptoms.

## 5. Conclusion

This study highlights the significant prevalence and impact of PMS among adult females in Sharjah, UAE, with 93% of participants experiencing at least one PMS symptom and a one-third prevalence rate. The findings revealed an overall proinflammatory dietary pattern among participants. Furthermore, a notable association exists between unfavorable dietary habits, as indicated by the DII, and the severity of PMS symptoms. Ultimately, this highlights the significance of diet in managing symptoms. To support enhanced QoL for women affected by PMS, it is recommended that dietary changes be implemented, focusing on increasing the intake of anti-inflammatory foods such as olive oil, nuts, fruits, vegetables, and certain herbal supplements. This approach may help mitigate the inflammatory responses associated with PMS and contribute to improved health outcomes for women during their childbearing years. Further research using consistent protocols and procedures to minimize the risk of bias is warranted to deepen the understanding of these relationships and optimize dietary recommendations for improving PMS-related psychological symptoms.

## Figures and Tables

**Table 1:** Participants' sociodemographic characteristics and anthropometric measurements (*N* = 305).

Variable	Mean ± SD	
Age (years)	23.56 ± 7.81	
Height (cm)	160.08 ± 6.46	
Weight (kg)	61.83 ± 14.29	
BMI (kg/m^2^)	24.09 ± 5.24	

**Variable**	***n***	**%**

Marital status		
Unmarried	264	86.6
Married	33	10.8
Divorced	7	2.3
Widowed	1	0.3
Education		
Primary	3	1.0
Secondary	10	3.3
High school	121	39.7
University	171	56.1
Nationality		
Local	134	43.9
GCC	21	6.9
Arab non-GCC	119	39.0
Non-Arabs	31	10.2
Waist circumference		
< 76 cm	185	60.7
> 76 cm	120	39.3

Abbreviations: BMI = body mass index, GCC = Gulf Cooperation Council.

**Table 2:** Participants' lifestyle characteristics (*N* = 305).

Variable	*n*	%
Smoking		
Never	283	92.8
Former	5	1.6
Current	17	5.6
Daily physical activity		
No physical activity	46	15.1
Less than 30 min	158	51.8
More than 30 min	101	33.1
Daily sun exposure		
Less than 15 min	198	64.9
More than 15 min	107	35.1
Dressing		
Abaya	221	72.5
Western	84	27.5
Housing		
Apartment	125	41.0
Villa	180	59.0

**Table 3:** Participants' dietary intakes (*N* = 305).

Nutrient or food intake/day	Mean ± SD	Min.	Max.
Energy (kcal)	3540.81 ± 1919.67	185.88	10,937.39
Protein (g)	137.06 ± 77.69	1.74	374.67
Carbohydrate (g)	398.35 ± 212.48	27.13	1496.55
Total fat (g)	160.88 ± 93.34	6.34	458.99
Cholesterol (mg)	563.11 ± 385.38	0.30	2424.43
Saturated fat (g)	68.58 ± 38.92	2.12	214.23
MUFA (g)	55.73 ± 35.17	1.79	164.08
PUFA (g)	25.66 ± 15.10	0.50	91.88
Sodium (mg)	3804.15 ± 2211.87	121.42	12,575.67
Vitamin C (mg)	295.50 ± 176.60	3.15	1603.76
Calcium (mg)	1778.32 ± 1038.41	31.91	5876.19
Iron (mg)	27.23 ± 16.11	0.57	115.16
Vitamin D (μg)	12.68 ± 9.40	0.00	56.63
Vitamin E (mg)	15.00 ± 9.18	0.40	49.12
Thiamin (B1) (mg)	4.78 ± 2.71	0.06	22.59
Riboflavin (B2) (mg)	10.88 ± 6.39	0.03	54.93
Pyridoxine (B6) (mg)	3.48 ± 2.08	0.14	11.86
Folate (B9) (μg)	529.15 ± 349.56	11.05	2191.52
Cobalamin (B_12_) (μg)	10.49 ± 14.73	0.02	149.35
Dietary fiber, total (g)	32.93 ± 22.26	1.47	127.56
Total sugar (g)	140.89 ± 77.21	11.84	530.54
Vitamin A (RAE) (μg)	2029.45 ± 1822.46	1.17	15,941.38
Rosemary (g)	2.46 ± 2.78	0.08	15.00
Thyme/oregano (g)	1.98 ± 2.74	0.08	20.00
Garlic (g)	4.35 ± 3.40	0.08	20.00
Onion (g)	11.53 ± 8.93	0.50	45.00
Black pepper (g)	3.97 ± 3.01	0.08	15.00
Turmeric (g)	3.01 ± 2.85	0.08	12.50
Saffron (g)	2.14 ± 3.23	0.08	25.00
Ginger (g)	2.48 ± 2.60	0.08	15.00

**Table 4:** Prevalence of premenstrual syndrome symptoms by the level of severity (N = 305).

Symptom	Never	Mild	Moderate	Severe
Depressed mood	57 (18.7%)	136 (44.6%)	90 (29.5%)	22 (7.2%)
Hopelessness	105 (34.4%)	115 (37.7%)	68 (22.3%	17 (5.6%)
Guilt feeling	105 (34.4%)	84 (27.5%)	92 (30.2%)	24 (7.9%)
Anxiety/worry	38 (12.5%)	108 (35.4%)	114 (37.4%)	45 (14.8%)
Affective lability	83 (27.2%)	107 (35.1%)	85 (27.9%)	30 (9.8%)
Increased sensitivity toward others	43 (14.1%)	116 (38.0%)	106 (34.8%)	40 (13.1%)
Anger feelings	41 (13.4%)	119 (39.0%)	91 (29.8%)	54 (17.7%)
Easily irritated/agitated	40 (13.1%)	114 (37.4%)	109 (35.7%)	42 (13.8%)
Lack of interest	67 (22.0%)	120 (39.3%)	92 (30.2%)	26 (8.5%)
Difficulty concentrating	60 (19.7%)	124 (40.7%)	88 (28.9%)	33 (10.8%)
Loss of control	93 (30.5%)	117 (38.4%)	77 (25.2%)	18 (5.9%)
Feeling overwhelmed	43 (14.1%)	99 (32.5%)	117 (38.4%)	58 (19.0%)
Lethargy/fatigue/decreased energy	25 (8.2%)	102 (33.4%)	120 (39.3%)	58 (19.0%)
Increased appetite	72 (23.6%)	103 (33.8%)	92 (30.2%)	38 (12.5%)
Craving certain foods	50 (16.4%)	110 (36.1%)	91 (29.8%)	54 (17.7%)
Hypersomnia	89 (29.2%)	113 (37.0%)	72 (23.6%)	31 (10.2%)
Insomnia	112 (36.7%)	96 (31.5%)	66 (21.6%)	31 (10.2%)
Breast tenderness	126 (41.3%)	97 (31.8%)	65 (21.3%)	17 (5.6%)
Breast engorgement or weight gain	114 (37.4%)	102 (33.4%)	73 (23.9%)	16 (5.2%)
Headache	67 (22.0%)	118 (38.7%)	90 (29.5%)	30 (9.8%)
Muscle, joint, abdominal, and back pain	37 (12.1%)	107 (35.1%)	97 (31.8%)	64 (21.0%)
Acne	63 (20.7%)	142 (46.6%)	75 (24.6%)	25 (8.2%)
Symptoms interfering with relationships	126 (41.3%)	86 (28.2%)	75 (24.6%)	18 (5.9%)
Symptoms interfering with work or school	101 (33.1%)	119 (39.0%)	68 (22.3%)	17 (5.6%)
Symptoms interfering with daily routine	91 (29.8%)	118 (38.7%)	76 (24.9%)	20 (6.6%)
Cumulative psychological symptoms	23 (7.5%)	168 (55.1%)	111 (36.4%)	3 (1.0%)
Cumulative physiological symptoms	11 (3.6%)	193 (63.3%)	97 (31.8%)	4 (1.3%)
Cumulative behavioral symptoms	92 (30.2%)	130 (42.6%)	75 (24.6%)	8 (2.6%)
Total PMS symptoms	**28 (9.2%)**	**199 (65.2%)**	**77 (25.2%)**	**1 (0.3%)**

*Note:* The bold values indicate the percent of participants who have all of the PMS symptoms.

**Table 5:** Odds ratios (ORs) and confidence intervals (CIs) for tertiles of the energy-adjusted dietary inflammatory index associated with the diet of patients with PMS.

Tertiles of the energy-adjusted dietary inflammatory index (E-DII)				
	**Tertile 1**	**Tertile 2**	**Tertile 3**	**E-DII (continuous)**

E-DII range	≤ −4.719	−4.720 to 15.756	≥ 15.760	305/305
PMS	102/305	102/305	101/305	
Multivariate adjusted^∗^	1.0 (ref)	1.77 (0.425–1.418)	2.151 (0.831–5.564)	1.639 (−0.107–0.121)
*p* value	*p*=0.001^∗^			NS

*Note:* Significant *p* value, because it is less than 0.05.

Abbreviations: E-DII = energy-adjusted dietary inflammatory index, OR = odds ratios, and NS = not significant.

^∗^Adjustments based on age, BMI, marital status, educational attainment, and nationality.

## Data Availability

The dataset used and analyzed during the present study is available from the corresponding author upon reasonable request.

## Nomenclature

APMSS: Arabic Premenstrual Syndrome Scale
BIA: Bioelectrical impedance
BFP: Body fat percentage
BMI: Body mass index
CI: Confidence interval
DSM–IV: Diagnostic and Statistical Manual of Mental Disorders, Fourth Edition
OR: Odds ratio
PMS: Premenstrual syndrome
UAE: United Arab Emirates
UOS: University of Sharjah
